# Assessment of the effect of insecticide-treated nets and indoor residual spraying for malaria control in three rural *kebeles* of Adami Tulu District, South Central Ethiopia

**DOI:** 10.1186/1475-2875-11-127

**Published:** 2012-04-25

**Authors:** Damtew Bekele, Yeshambel Belyhun, Beyene Petros, Wakgari Deressa

**Affiliations:** 1 Natural and Computational Sciences, Biology Department, Debre Markos University, Debre Markos, Ethiopia; 2 School of Biomedical and Laboratory Sciences, College of Medicine and Health Sciences, University of Gondar, Gondar, P.O. Box 196, Ethiopia; 3 Life Science faculty, Microbial Cellular and Molecular Biology unit, Biomedical Sciences, Addis Ababa University, Addis Ababa, Ethiopia; 4 School of Public Health, Epidemiology and Biostatics unit, Addis Ababa University, Addis Ababa, Ethiopia

## Abstract

**Background:**

In the Adami Tulu District, indoor residual spraying (IRS) and insecticide-treated nets (ITNs) has been the main tool used to control malaria. The purpose of this study was to assess the effect of IRS and ITNs control strategies in Aneno Shisho *kebele* (lowest administrative unit of Ethiopia) compared with Kamo Gerbi (supplied ITN only) and Jela Aluto (no IRS and ITNs), with regards to the prevalence of malaria and mosquito density.

**Methods:**

Cross-sectional surveys were conducted after heavy rains (October/November, 2006) and during the sporadic rains (April, 2007) in the three *kebeles* of Adami Tulu District. Malaria infection was measured by means of thick and thin film. Monthly collection of adult mosquitoes from October-December 2006 and April-May 2007 and sporozoite enzyme-linked immunosorbent assay (ELISA) on the collected mosquitoes were detected. Data related to the knowledge of mode of malaria transmission and its control measures were collected. Data collected on parasitological and knowledge, attitude and practice (KAP) surveys were managed and analysed using a statistical computer program SPSS version 13.0. A P-value <0.05 was considered to be statistically significant.

**Results:**

The overall prevalence of malaria was 8.6% in Jela Aluto, 4.4% in Kamo Gerbi and 1.3% in Aneno Shisho in the two season surveys. The vector, *Anopheles gambiae* s.l., *Anopheles pharoensis* and *Anopheles coustani* were recorded. However, sporozoite ELISA on mosquito collections detected no infection. The difference in overall malaria prevalence and mosquito density between the three *kebeles* was significant (P<0.05).

**Conclusions:**

The present study has provided some evidence for the success of ITNs/IRS combined malaria control measures in Aneno Shisho *kebele* in Adami Tulu District. Therefore, the combined ITNs/IRS malaria control measures must be expanded to cover all *kebeles* in the District of Ethiopia**.**

## Background

Malaria is one of the serious tropical diseases caused by protozoan parasites transmitted by the bite of female *Anopheles* mosquitoes. Malaria control is an increasingly important focus for the international body concerned with public health and disease control. In Africa ITNs and IRS are both effective for malaria vector control. Insecticide-treated nets are being promoted throughout Africa as a fundamental preventive strategy to Roll Back Malaria [1]. However, the control of malaria in Africa is less successful because of the occurrence of drug resistant parasites and insecticide resistant vectors [2].

Compared with placebo treated, the wide use of ITNs was shown to reduce incidence and provide significant protection against mortality attributed to malaria, clinical attacks of malaria and malaria infection [3]. In addition, in a small field trial in a Kenyan school [4] reported a reduction of 97.3% in malaria attack for children who slept under bed nets.

Currently, there are a number of possible precipitating epidemic factors of malaria in addition to environmental or climatological factors, chloroquine-resistance of *Plasmodium falciparum*, high scale population movement (due to resettlement and labor forces in agro-industrial schemes) and deterioration of vector control operations [5].

The nationally-adopted malaria prevention and control strategies in Ethiopia include early diagnosis and prompt treatment, selective vector control strategy using ITNs and IRS [6]. Dichloro-diphenyl-trichloroethane (DDT) has been used in Ethiopia for nearly five decades and is still the insecticide of choice. Most spraying operations are carried out during June and July to prevent potential malaria epidemics that can occur during September to November after the heavy rains and in January and February to prevent transmission following the small rains [7]. Indoor residual spraying with DDT can be used for insecticides on the wall of dwellings up to six months [8]. However, the increase and spread of insecticide resistance, the high degree of replastering rate of sprayed houses in the context of expanded urbanization, and an increased number of natural resource development projects, has necessitated the utilization of all appropriate technological and management techniques in an integrated approach to bring about an effective degree of vector suppression [9].

Widespread use of ITNs by the entire community results in a decline in human-mosquito contact, a decrease in the number of mosquitoes, and a reduction in malaria transmission, which will lead to a decline in malaria-related morbidity and mortality [7]. In addition, ITNs provide protection against nuisance mosquitoes and kill head lice and bedbugs. A study carried out for the implementation of insecticide-treated mosquito nets in malaria control in Ethiopia showed that the community reported unaffordability of ITNs to most households and improper use of nets. However, acceptability and willingness to use ITNs for malaria prevention was very high [10].

The control of malaria and other vector borne diseases in Ethiopia until recently relied up on case treatment and vector control through application of water dispersible residual insecticides, such as DDT, to the interior surfaces of walls, ceilings and roofs of houses. Currently, in line with the strategies set by the Roll Back Malaria, wide-scale use of insecticide-treated nets is added to the existing control methods. The objective of this study was to assess the impact of ITNs/IRS combined malaria control strategies currently used in Aneno Shisho *kebele*, Adami Tulu District with regard to malaria transmission and on the *Anopheles* vector species.

## Methods

### Description of the study area and population

The study was conducted in Adami Tulu District, which is part of the East Showa Zone of the Oromia Regional State in between October 2006 and May 2007. Geographically the area is located between 3820 and 38.55 and 735and 805. The District covers an area of 1403.3km^2^ (Adami Tulu District agricultural development office report, 2006/07; unpublished report). Ecologically, Adami Tulu is found in the Central Rift Valley of Ethiopia and it is located at 160km away from Addis Ababa to the South Central part and the significant part of the main rift valley lake in the area is Lake Zeway. The relief of the area is characterized by plain and flat stretched land.

**Figure 1:**
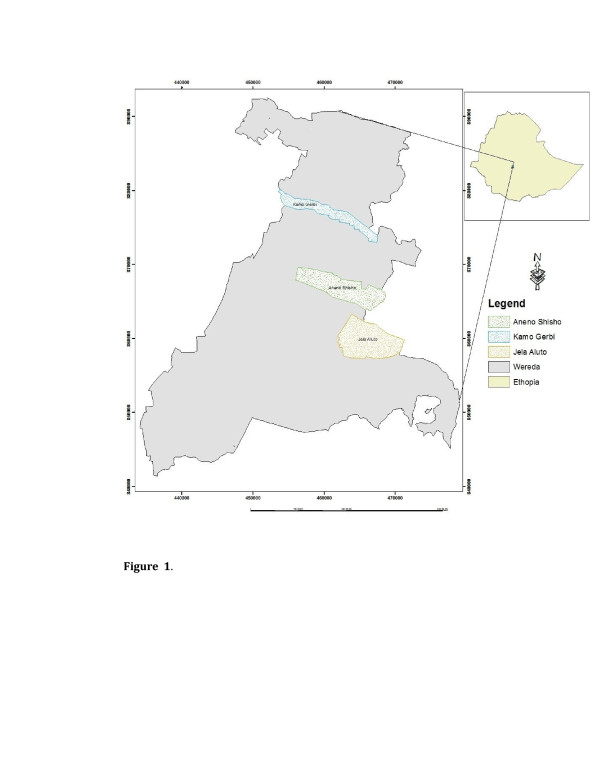
**Map of the Adami Tulu District and distribution of*Kebeles*including the study area: Jela Aluto, Kamo Gerbi and Aneno Shisho, in 2006/07.**

A study was undertaken for five months during the major and minor malaria transmission seasons in Aneno Shisho, Kamo Gerbi and Jela Aluto *kebeles* in the Adami Tulu District. The distance between Aneno Shisho and Kamo Gerbi was 14km, and between Aneno Shisho and Jela Aluto 12km (Figure 1). These *kebeles* were mainly selected due to the fact that there was a major malaria epidemic in the area since in 1992 (the area, with altitudes ranging from 1,600-1,700 metres, is epidemic prone) [11]. The other reason is about four decades before the time of this study, detailed entomological studies had been conducted in the area and both *Anopheles gambiae* s.l. and *Anopheles pharoensis* are known to exist in the area, hence these could be used as baseline information for undergoing this study.

Aneno Shisho has an average altitude of 1,660m above sea level (a.s.l), Kamo Gerbi and Jela Aluto have average altitude of 1,680ma.s.l. and 1,675ma.s.l., respectively. Among the three *kebeles*; Aneno Shisho received DDT and ITNs (population, 4179), Kamo Gerbi received ITNs only (population, 4100), and Jela Aluto received neither of the DDT and ITNs (population, 4286). Their total population was 12,565, according to a census taken in 1994 (Central Statistical Authority). At each of the three *kebeles*, the major occupation of the population was dependent on agriculture and livestock herding. Most houses in the three *kebeles* were circular *tukuls* with thatched conical roofs and mud or thatch walls. There are a few rectangular houses with the roofs of corrugated metal sheets. Cattles were usually kept in outdoor enclosure made of a loose framework of pests and twigs near the human dwellings and it was observed as the breeding site for the mosquitoes.

The residual treatment applied in Aneno Shisho were DDT 75% water dispersible powder (WDP) used in a spray pump with capacity of eight liters of liquid to obtain the target dosage of 2 gm/m^2^. Spraying of DDT has been carried out in Aneno Shisho beginning from July 2005 and the second round spraying has been carried out in January 2006. Also, DDT has been used in July 2006 and January 2007, as its residual life span is six months. In Aneno Shisho and Kamo Gerbi an average of two LLINs (PermaNets) per household were first supplied free of cost in January 2005 by Adami Tulu health center. The PermaNet supplied to the inhabitants of Aneno Shisho and Kamo Gerbi was ready-impregnated. It is made of polyester netting material (mesh 25 holes/cm^2^ with deltamethrin incorporated with 55mg ai/m^2^) in a resin coating of the fibers [12], which remain effective for three years under normal use.

The area received an annual rainfall of about 4,232.3mm and mean annual rainfall was 705.3mm for six consecutive years (2000-2005). In the year from 2000-2005, mean monthly maximum temperature ranges from 26.3C to 29C, whereas mean monthly minimum temperature ranges from 12.1C to 16.4C (Source: National Meteorological Services Agency of Ethiopia, unpublished data).

The area, like most part of Ethiopia, has two periods of rainfall, June-September, known as the heavy rains and March-April, the small rains. Malaria is the principal cause of morbidity in the area affecting all age groups. The malaria control center of Adami Tulu District is responsible for most case management of uncomplicated malaria, vector-control activities and malaria epidemic control in the area. The major control measures at the time of the survey consisted of indoor residual spraying such as DDT, insecticide-treated nets, anti-malarial drug administration distribution to confirmed cases, but sometimes for presumptive treatment also.

### Indoor residual spraying (IRS)

Indoor residual insecticides with DDT have been applied for nearly five decades in Aneno Shisho *kebele* of Adami Tulu District (Ato Haile Gebre - head of malaria control center of Adami Tulu District, Personal Communication). Residual spraying of DDT has been used in this area in the form of DDT 75% water dispersible powder (WDP). To obtain target dosage of 2 grams of insecticide per m^2^, 535 grams of DDT 75% WDP are used in a spray pump with capacity of eight liters of liquid and is sprayed from stirrup pumps or hand-compression sprayers. Dichloro-diphenyl-trichloroethane spraying in Aneno Shisho at a dosage of 2g/m^2^ was carried out twice yearly on the walls and roofs of all houses and domestic animal shelters. The first spraying operations indoors are carried out in June and July, just before the major transmission season and the second round spray are also carried out in January and February, just before the minor transmission season of malaria. This application was carried out since DDT was the residual treatment that remains effective up to six months.

### Insecticide-treated bed nets (ITNs)

Promoting the use of insecticide-treated netting materials is one of the principal technical approaches of the Roll Back Malaria (RBM) partnership in the control of malaria. Therefore, in addition to DDT the use of ITNs is the range of malaria control strategies available. In January 2005, an average of two PemaNets per household has been delivered for Aneno Shisho and Kamo Gerbi *kebeles* free of charge by the Adami Tulu District health center. The nets provided are long-lasting insecticide-treated nets (LLINs) or PermaNets as they eliminate the need for net re-impregnation of conventional nets and is recommended by WHOPES (WHOs pesticide Evaluation Scheme at the Division of Control of Tropical Diseases) for Ethiopia for malaria prevention. This PermaNet is manufactured by Vestergaard Frandsen of Denmark that are treated with a higher dose of wash-resistant deltamethrin, which is said to remain effective for up to 20 washes, or for a period of three years under normal use.

### Sampling technique and sample size determination

The survey was conducted based on similar geographical areas and considering the flight range of *Anopheles*. Three *kebeles* (Aneno Shisho, Kamo Gerbi, and Jela Aluto) were selected. Among the three *kebeles*; Aneno Shisho received ITNs and IRS, Kamo Gerbi received ITNs only, and Jela Aluto received neither of the two malaria control measures.

To determine the required sample size for the prevalence study, a malaria prevalence of 39% is assumed. This is based on the result of unpublished report of Adami Tulu Health Centre records from 1999-2006. A sampling error (d) of 0.04 is considered to get a reasonable estimate. The sample size (n) was determined by using the formula for estimating single proportion, where level of significance is 0.05. Accordingly, the minimum sample size was determined to be 571. However, a total of 218 households were selected systematically from the three *kebeles*, by taking every 5^th^ household from a random start based on their registration list. Two-step sampling was performed to get the required sample size as the study units are the individuals of the selected households. During the first and second survey three individuals per household were sampled for the three *kebeles* to sample 654 individuals.

### Parasitological survey

A blood sample was taken from all selected members of households by experienced laboratory technician by pricking the finger with disposable blood lancet, and thick and thin blood smears were prepared on the same slide and identification numbers marked on the thin films. The thin films were fixed using 100% methanol and then all slides were stained with 3% Giemsa for 20 minutes. Parasite positivity was then determined from thick smear and species identification was carried out from thin smear slide preparations. Each sample was studied by two qualified laboratory technicians in Health Centre and confirmatory examination was carried out by the third technician in the Biomedical Laboratory of Biology Department, Addis Ababa University. During this survey was carried in the seasons of October/November 2006 and April 2007 a verbal consent was obtained for taking blood film from adult participants in sampled households and appropriate anti-malarial treatment was given to positive cases.

### Adult mosquito collection

Adult female mosquito collections were made using aspirators and CDC light-trap from indoors and outdoors. Indoor resting mosquito collections from human dwellings were conducted monthly in the three *kebeles* for five consecutive morning hours (6:00-8:30) between October-December 2006 and April-May 2007. The collections were made by the experienced entomology technician from the local malaria control center and by the investigator of the study in using flash lights and an aspirator and aspirating Anopheline mosquitoes from walls, ceilings and other objects. Mosquitoes were also collected by aspirator from outdoor resting sites (cattle sheds, pit shelters and tree holes). Two dry cell battery-operated CDC light-traps were set indoors in the three sites and operated from 18:00 to 06:00 hours to collect adult mosquitoes. The collected mosquitoes were placed in paper cups and delivered to the temporary examination post in the field. This survey was done by a house-to-house visit with a view to collecting blood samples, *Anopheles* mosquito collection and gathering demographic information from the members of the households selected with the help of a questionnaire.

### Mosquito identification and sporozoite detection

Identification of all adult mosquitoes collected was undertaken using a key [13]. Dried mosquitoes were kept in vials containing silica jell desiccant and ELISA were developed to detect *P. falciparum* and *Plasmodium vivax* circumsporozoite proteins in malaria-infected mosquitoes [14]. Anopheline mosquito collection and identification was undertaken by an investigator of the study with an entomology technician from Adami Tulu malaria control center. The detection of sporozoite rates was conducted by the investigator of the study under supervision of a senior entomology technician from the Institute of Ethiopian Health and Nutrition Research.

### Knowledge, attitude and practice (KAP) surveys

Malaria control data were collected from February to April, 2007 at the three *kebeles* of Adami Tulu District using semi-structured interview administered in the local language (Afaan Oromo). A total of 218 household heads as informants were selected systematically from their registration list of each *kebele* by taking every 5^th^ household from a random start. Of these, informants from Jela Aluto (73), Kamo Gerbi (72), and Aneno Shisho (73) were selected. The semi-structured interview guide is comprised of open-ended and closed questions, and data were obtained on informants knowledge, attitudes and practices regarding the mode of malaria transmission and its prevention and control in the local population.

### Ethical considerations

At the beginning of the study, the purpose of the investigation was explained to administrative staff at District and Kebele levels and requested to cooperate. Similarly, from each of household heads, who was selected as representative sample was also asked to consent verbally and participated in the study. Blood smear was obtained with finger prick using disposable blood lancet and cotton immersed in 75% alcohol. During the study period, any *kebele* inhabitant found sick of malaria was given treatment in collaboration with Adami Tulu District Health Post freely. Approval to conduct the study was granted by the ethical committee in the Department of Biology, Addis Ababa University.

### Data analysis

Data collected on parasitological and KAP surveys were managed and analysed using a statistical computer program SPSS version 13.0. The comparisons between *kebeles* in malaria prevalence, mosquito density and mode of malaria transmission were carried out using the Pearsons chi-square test. A P- value <0.05 was considered to be statistically significant.

## Results

### Malaria prevalence in the study *kebeles* of Adami Tulu District

Of the total 720 individuals enrolled in the study, 337 (46.8%) were males and 383 (53.2%) females. Malaria positive individuals were identified from the three *kebeles* in October/November 2006 and April 2007. Out of the total 1429 blood films examined after the heavy and small rains, 68(4.8%) were found to be positive. The parasite species composition showed 88.2% *P. vivax* and 11.8% *P. falciparum*. The malaria prevalence rate in October/November 2006 in Jela Aluto, which did not covered by ITNs or IRS was significantly higher (10.4%) than Kamo Gerbi which was covered by ITN (5.4%) whereas in Aneno Shisho (1.7%), which was covered both by ITNs and IRS. The total *Plasmodium* prevalence was significantly different among the three *kebeles* (P<0.05).

**Figure 2:**
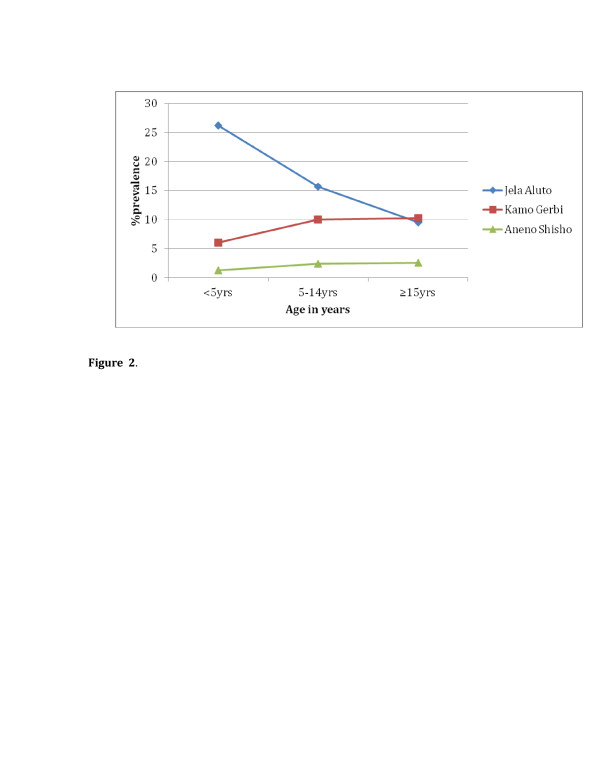
The prevalence of malaria infection by age in the study areas in Adami Tulu District, Oct/Nov 2006 and April 2007.

In the under five years of age, malaria prevalence of October/November 2006 and April 2007 was 26.2% in Jela Aluto; 6.0% in Kamo Gerbi and 1.2% in Aneno Shisho as indicated in Figure 2. The difference in the under five years of age in prevalence among the three *kebeles* was statistically significant (P<0.05). On the other hand, malaria prevalence in the age group above 15years was 9.5% in Jela Aluto; 10.2% in Kamo Gerbi and 3.7% in Aneno Shisho. The reduction in children under five years of age in parasite rate in the protected village of Kamo Gerbi and Aneno Shisho was due to ITN protection level (51%) and (64.4%), respectively in relation to older as observed from the KAP study.

#### Adult mosquito survey

**Figure 3:**
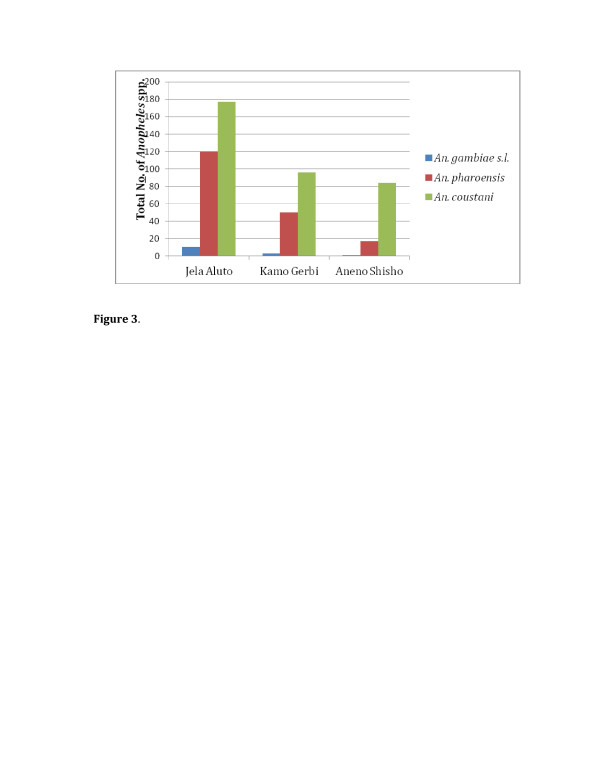
**Total adult*Anophelines*collected monthly from the three study*Kebeles*in Adami Tulu District, Oct/Nov 2006 and April/may 2007.** Legend: *Whereas only one *An. wellcomei* collected from Kamo Gerbi.

Four species of anopheline mosquitoes *An. gambiae s.l., An. pharoensis, Anopheles coustani* and *Anopheles wellcomei* were collected and identified. The finding showed *An. coustani* and *An. pharoensis* were found to be the most abundant, comprising 63.8% and 33.4%, respectively. However, the *An. gambiae s.l.* and *An. wellcomei* were less abundant (Figure 3).

The densities of *An. gambiae s.l.* were 11(73.3%) in Jela Aluto, 3(20%) in Kamo Gerbi and 1(6.6%) in Aneno Shisho. In addition, *An. pharoensis* and *An. coustani* were highest in the untreated *kebele* (Jela Aluto), where it reached 64.2% and 49.6%, respectively. Whereas in Kamo Gerbi 26.7% and 26.9%; and in Aneno Shisho 9.1% and 23.5% (Figure 3).

#### Sporozoite rate

The sporozoite detection with ELISA test was carried out on dried and desiccated mosquitoes. Out of a total of 380 specimens (15 *An. gambiae s.l.*, 187 *An. pharoensis* and 178 *An. coustani*) tested for the presence of *P. falciparum* and *P. vivax* sporozoite antigens in the head-thorax region by the ELISA method and none were found infected.

#### Knowledge, attitude and practice (KAP) survey about mode of malaria transmission and its control measures

**Figure 4:**
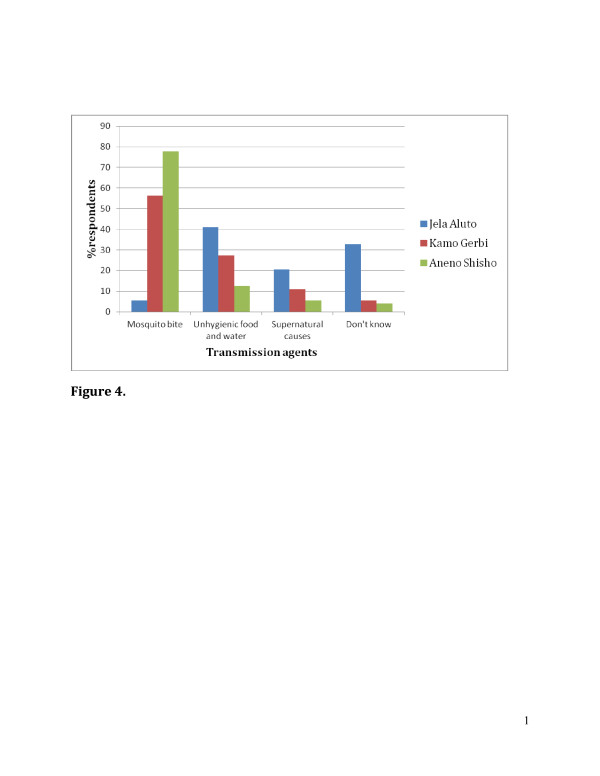
Knowledge, attitude and practice (KAP) survey about mode of malaria transmission and its control measures on three study Kebeles in Adami Tulu District, 2006/07.

The study population included 218 individuals, 119 (54.6%) female and 99 (45.4%) male. The mean age was 40.8years with a range of 18-75years. Farmer was the most commonly reported occupation (82.6%) followed by daily labor and merchant accounts for 13.3% and 4.1%, respectively. In regard to the knowledge of malaria transmission, in Jela Aluto only 5.5% of respondents stated that the mode of malaria transmission is by mosquito bite, whereas, in Kamo Gerbi 56.2% and in Aneno Shisho 77.8% respond the exact way of malaria transmission. Those that responded dont know in Jela Aluto accounted for 32.9% and the rest ways of transmission respond by the communities were presented (Figure 4).

Concerning the malaria control measures in the three *kebeles*; 41.1% of respondents in Aneno Shisho replied by using insecticide spray; 12.3% and 9.7% of respondents in Kamo Gerbi and Jela Aluto, respectively replied also by using IRS. In addition, 52.1%, 63% and 11.1% of respondents from Aneno Shisho, Kamo Gerbi and Jela Aluto, respectively suggested using ITNs. While 22.2% in Jela Aluto responded do not know.

## Discussion

The overall prevalence of malaria in the first survey (October/November 2006) and second survey (April 2007) was 8.6% in Jela Aluto, 4.4% in Kamo Gerbi and 1.3% in Aneno Shisho. This data was found during no malaria control under taken in Jela Aluto, while there was ITN in use in Kamo Gerbi and both ITN and IRS in use in Aneno Shisho. Regarding elevations among the three *kebeles*, there were no significant differences. In connection with this in Ethiopia areas below 2,000 metres altitude are considered malarious. However, when microclimate and weather conditions are favourable, malaria transmission is reported to occur in areas, higher than 2,000 metres above sea level [15]. Also, the distances among the three *kebeles* were far from each other as indicated in Figure 1, and then there is no *Anopheles* flight. People also confined to their own *kebeles* for their subsistence agriculture and cattle herding. Furthermore, in each *kebeles* there were drainage ditches, swamps, hoof prints, and cattle sheds, which have contributed to the creation of breeding areas. Therefore, the significant difference in the prevalence among the three *kebeles* was related to the introduction of ITNs and IRS that had direct impact on the malaria transmission. The highest prevalence of malaria was detected in <5years of children in Jela aluto (26.2%), followed by Kamo Gerbi (6%) and Aneno Shisho (1.2%) as shown in Figure 2. This could be associated with the ITN and IRS coverage in Aneno Shisho, ITN coverage in Kamo Gerbi and neither of the two malaria control measures in Jela Aluto as obtained from the KAP survey.

As the survey undertaken the dwellings of Aneno Shisho were sprayed with DDT in June and July 2006, just before the major transmission season and for the second round in January and February 2007, just before the minor transmission season through health center of Adami Tulu District. In connection with this, in Africa IRS has been focused at selected ecological zones such as those experiencing unstable transmission [16]. Aneno Shisho and Kamo Gerbi *kebeles* had received two PermaNets per household without any cost disseminated through health center of Adami Tulu District. During this study conducted in the District between October-December 2006 and April-May 2007, the total density of *An. gambiae s.l., An. pharoensis* and *An. coustani* collected from the three study *kebeles* were 15(2.7%), 187(33.4%) and 357(63.8%), respectively (Figure 3). Treated-mosquito nets have been shown to provide strong protection against malaria and are currently widely applied as a vector control measure particularly in Sub-Saharan Africa [17]. Similarly, assessment of the effects of ITNs and IRS in malaria control showed the importance of implementation of ITNs and insecticide spraying as a malaria control strategy in malarious areas of Adami Tulu District.

In relation to the relative density of *Anopheles* species Oct/Nov 2006, the malaria prevalence was 8.6% in Jela Aluto where there was no malaria control; on the other hand in Kamo Gerbi and Aneno Shisho *kebeles* malaria prevalence in Oct/Nov 2006 to April 2007 was reduced in comparison with the corresponding period in Jela Aluto. The findings obtained from studies carried out in the same District revealed that the prevalence of *P. falciparum* and *P. vivax* were 30.2% and 6.2%, respectively [18]. The use of ITNs is suggestive of the fact that reduction of man-vector contact and significantly reduced the transmission of malaria in the area.

The use of insecticide-treated bed nets was recommended for the area as an option for reducing contact with *Anopheles arabiensis*[11]. This was based on the experience of pyrethroid-treated bed net that has been shown to be an effective tool in the reduction of malaria mortality and morbidity in some countries, and had reduced malaria transmission with large-scale use [19]. On the other hand, in a comparative study done in neighbouring Kenya showed that sleeping under an ITN reduced the risk of infection by 63% and sleeping in a room sprayed with insecticide reduced the risk by 75% [20]. This study recommends the use of IRS as a more effective and cheaper option in communities with low and seasonal risks of infection.

This is suggestive of the use of insecticide-treated bed nets can be considered an option for reducing contact with *An. gambiae s.l.,* but would be ineffective against *An. pharoensis,* as it has been shown that most man-vector contact with this species occurs outdoors during the early hours of the evening [11]. The average density of *Anopheles* mosquito species in the insecticide-treated *kebeles* of Aneno Shisho and Kamo Gerbi was lower than the density in the untreated Jela Aluto area. The difference can be attributed to the insecticide malaria control.

The density of *An. gambiae s.l.* and *An. pharoensis* were higher from October to December than from April to May. These results indicated the similarity with the national pattern of malaria transmission where high number of malaria cases occurs during the major transmission season from September through December following the heavy rains [21]. It has been reported that *An. arabiensis* fed predominantly indoors than outdoors [3]. Furthermore, the predominantly endophagic behaviour of *An. arabiensis* had reported in different African countries including Ethiopia [22]. From the results of this study area, the use of insecticide-treated bed nets can be considered effective for reducing man-vector contact with *An. gambiae s.l.*

The exophagic behaviour of *An. pharoensis* has been documented in Zeway [11]. Furthermore, a study in Gambella, Ethiopia had also shown this species to be feeding indoors and outdoors in equal proportions [23] indicating eco-geographic variations in its feeding behaviour. *Anopheles pharoensis* may play significant role in the transmission of malaria during the dry season as suggested by its higher density that tends to increase whereby the density of *An. gambiae s.l.* is known to drop. That is, *An. pharoensis* may take the role of main malaria vector during the dry season [11]. *Anopheles coustani* which was collected from outdoors (cattle sheds) was previously reported as common in Akaki, out district of Addis Ababa [24].

Out of a total of 380 specimens (15 *An. gambiae s.l.*, 187 *An. pharoensis* and 178 *An. coustani*) tested for the presence of *P. falciparum* and *P. vivax* sporozoite. The sporozoite ELISA did not demonstrated infection; this finding is coincided with the study carried out in the same District that from a total of 334 *An. arabiensis* and 272 *An. pharoensis* tested for the sporozoite antigens; none were found to be infected [11]. In contrast, 262 *An. gambiae* s.l. and 436 *An. pharoensis* were assayed by ELISA. Of these sporozoite rates of 0.76% (*P. falciparum*) for *An. gambiae s.l.* and 0.47% (*P. vivax*) for *An. pharoensis* were reported in the Gambella area of western Ethiopia (19). Moreover, malaria sporozoite rates were determined by ELISA, out of *An. arabiensis*, 0.5% infective with *P. falciparum* and 1.76% with *P. vivax* were found in Southern Ethiopia from Sille [25]. Therefore, although the sporozoite ELISA did not demonstrated infection, their abundance, which coincided with increasing malaria prevalence in this study area, may indirectly support the role of these species as malaria vectors at Adami Tulu District.

The significantly high abundance of adult *Anopheles* mosquito population in Jela Aluto compared to Kamo Gerbi and Aneno Shisho suggests that the use of ITN and/or insecticide spraying had reduced the indoor resting mosquito vectors. Thus, malaria control programs must also include vector control to reduce drug pressure in the endemic *kebele*. In particular, insecticide-impregnated bed nets were found to substantially reduce occurrence of malaria in the population, especially in the wet season in Ghana [26]. Moreover, insecticides repel mosquitoes and by so doing reduce the number of mosquitoes entering the sprayed room [27]. In contrast to the present study, insecticide-treated nets and insecticide sprays have been shown to affect the indoor resting habit of mosquitoes by increasing the rate of exophily [28]. This was also shown in Ethiopia from Gambella [23].

The combined effect of IRS and ITN that reduces mosquito human contact was directly associated with low malaria prevalence in the present study. A comparable finding was reported from Eritrea whereby a combination of IRS, larvacidal measures and malaria case management was used to combat malaria [29]. It was noted that the drop in infection rates was closely related to the amount of DDT used and the number of insecticide-treated bed nets distributed.

In the present study, the ITN/IRS control measures did not fully control malaria transmission as low level prevalence were registered albeit in a significantly lower level in the ITN/IRS *kebele*. The reason for this may be due to inadequate coverage of households with ITNs whereby each household received only two PermaNets and only children slept inside the nets in majority of the cases, leaving the adults exposed to high risk of infection. This was reflected by the relatively higher malaria prevalence in adults in the *kebeles* that received ITNs. Furthermore, insecticide resistance in the region can be the cause for the relatively high *Anopheles* catch as it was indicated [11], which would maintain malaria transmission in the study locations.

In conclusion, based on the findings of the study, the prevalence of malaria and the number of Anopheline mosquito species was significantly higher in the *kebeles* of Adami Tulu District where no malaria control measures were in use. Therefore, the concurrent use of ITNs and IRS was a much more effective malaria control measure. In order to have adequate knowledge about the mode of malaria transmission health education is very important in addition to disseminating ITNs and/or insecticide spray since the level of awareness was not very high even in those communities that received the control interventions.

## Abbreviations

a.s.l. = Above sea level; DDT = Dichloro-diphenyl-trichloroethane; ELISA = Enzyme-linked immunosorbent assay; IRS = Indoor residual spraying; ITNs = Insecticide-treated nets; KAP = Knowledge, attitude and practice; LLINs = Long-lasting insecticide-treated nets; MOH = Ministry of Health; RBM = Roll Back Malaria; WDP = Water-dispersible powder; WHO = World Health Organization.

## **Competing interests**

All authors have read the manuscript and declared that no competing interests exist.

## Authors contributions

BD participated to the study design, undertook the field study, participated in the data collection, analysis and interpretation, and drafted the manuscript. BY involved in the statistical analysis and interpretation, drafted and revised the manuscript and has given approval of the version to be published. PB designed the study, participated in the collection of the data and helped to draft the manuscript. DW participated in the design of the surveys, and coordinated the draft of the manuscript. All authors read and approved the final manuscript.
